# Sepsis: Changing Definitions, Unchanging Treatment

**DOI:** 10.3389/fped.2018.00425

**Published:** 2019-01-23

**Authors:** Nchafatso Gikenyi Obonyo, Luregn Jan Schlapbach, John Francis Fraser

**Affiliations:** ^1^Initiative to Develop African Research Leaders (IDeAL-DELTAS), Kilifi, Kenya; ^2^KEMRI-Wellcome Trust Research Programme, CGMRC, Kilifi, Kenya; ^3^Critical Care Research Group, The Prince Charles Hospital, Chermside, QLD, Australia; ^4^Wellcome Trust Centre for Global Health Research, Imperial College London, London, United Kingdom; ^5^Paediatric Critical Care Research Group, Child Health Research Centre, The University of Queensland, South Brisbane, QLD, Australia; ^6^Paediatric Intensive Care Unit, Queensland Children's Hospital, South Brisbane, QLD, Australia; ^7^Department of Pediatrics, Inselspital Universitätsspital Bern, Bern, Switzerland; ^8^Faculty of Medicine, The University of Queensland, Herston, QLD, Australia; ^9^School of Medicine, Griffith University, Gold Coast, QLD, Australia

**Keywords:** sepsis, septic shock, definitions, pediatric populations, treatment bundles

## Abstract

The recently revised Sepsis-3 definitions were based on criteria that were derived and validated in adult patient databases from high income countries. Both sepsis and septic shock continue to account for a substantial proportion of mortality globally, especially amongst children in low-and-middle income country settings. It is therefore urgent to develop and validate standardized criteria for sepsis that can be applied to pediatric populations in different settings, including in- and outside intensive care, both in high- and low/middle- income countries. This will be a pre-requisite to evaluate the impact of sepsis treatment strategies to improve clinical outcomes.

## Background

In 2016, the International Sepsis Definition Taskforce convened by the Society of Critical Care Medicine, and the European Society of Intensive Care Medicine, updated definitions and clinical criteria for sepsis. These should facilitate recognition, targeted management of patients with sepsis and also improve accurate characterization of the global sepsis burden (1). Sepsis-3 defines sepsis as life-threatening organ dysfunction caused by a dysregulated host response to infection, while the concept of septic shock incorporates profound circulatory, cellular and metabolic abnormalities associated with a greater risk of mortality (1). These new Sepsis-3 criteria reflect advances made in the understanding of the pathobiology, epidemiology, and management of sepsis. While the concept underlying the new sepsis definition can be applied to all age groups, the operationalization of definition was derived and validated in adult cohorts only.

In May, 2017, the World Health Assembly (WHA), the World Health Organization's (WHO) decision-making body, adopted a resolution recognizing the need to improve the prevention, diagnosis, and management of sepsis as a priority (2). It is currently estimated that 30 million cases and 6 million sepsis-related deaths occur worldwide each year including 3 million newborns and 1.2 million children who suffer from sepsis globally on an annual basis (3). Translating the WHO resolution at national and international level into actions leading to improved outcomes for children will require addressing the unique features characterizing epidemiology, host responses, and outcomes (4) to ensure accurate definition, and targeted treatment.

## Definitions of Pediatric Sepsis

The Sepsis-3 criteria were based on systematic reviews, Delphi processes, and stringent methodology to develop and validate robust criteria for sepsis (1, 5, 6), and the merit in this data-driven approach is widely recognized. Yet, validity remains restricted to the adult populations in which the criteria were developed and tested, and a gap exists in relation to pediatric sepsis. Several studies have demonstrated that the application of Sepsis-3 derived criteria to children in intensive care settings in high income countries performs reasonably well (7, 8). However, a number of challenges remain to be addressed to translate these to settings outside intensive care, including emergency departments, and in particular low-and-middle income (LMIC) settings (3). In the global context, pediatric sepsis burden occurs disproportionately in LMICs with a devastating impact on neonatal and childhood mortality (2). However, robust data on the burden of pediatric sepsis in LMICs remain scarce (3). Currently, there is no definition of pediatric sepsis that is harmonized with Sepsis-3, a shortcoming recognized by the 2016 Sepsis International Consensus Taskforce which acknowledged the need to develop similar definitions for pediatric populations, incorporating clinical criteria that take age-dependent variation into account (9). Presently used clinical criteria for diagnosing sepsis in children in LMICs include the 2005 Pediatric Sepsis Consensus Conference (PSCC) (10, 11) and the World Health Organization's Integrated Management of Childhood Illnesses (WHO-IMCI) (12). Further criteria facilitating assessment of septic shock in neonates and children were proposed by the American College of Critical Care Medicine (ACCM) in 2002 and subsequently updated in 2007 (13) and 2014 (14). These have also been applied in LMICs settings but to varied extents due to limitations in ability to implement criteria such as inotrope therapy as well as intensive care hemodynamic monitoring and support. Box 1 below compares and contrasts different criteria used to identify pediatric sepsis. Of note, subtle but substantial differences exist in some of the cut-off values for various variables used in defining sepsis when comparing the PSCC, WHO-IMCI, and ACCM criteria (Table 1).

Box 1 **Criteria to recognize sepsis and septic shock in children.**

**Pediatric Sepsis Consensus Conference definitions**

The 2005 pediatric Sepsis Consensus Conference, PSCC, definition of sepsis is systemic inflammatory response syndrome (SIRS) in the presence of, or as a result of, suspected or proven infection, whereby SIRS comprises temperature dysregulation (defined as core body temperature >38.5 or < 36°C); tachycardia (defined as a mean heart rate >2 SD above normal for age in the absence of external stimulus chronic drugs, or painful stimuli; or otherwise unexplained persistent elevation over a 0.5–4 h time period or for children < 1 year old); bradycardia (defined as a mean heart rate < 10th percentile for age in the absence of external vagal stimulus, βeta-blocker drugs, or congenital heart disease; or otherwise unexplained persistent depression over a 0.5 h time period); respiratory rate dysregulation (defined as a mean respiratory rate >2 SD above normal for age or mechanical ventilation for an acute process not related to underlying neuromuscular disease or the receipt of general anesthesia); leucocyte count elevated or depressed for age, or >10% immature neutrophils, but not secondary to chemotherapy-induced leukopenia (10, 11). Septic shock was defined as presence of sepsis and cardiovascular organ dysfunction in the PSCC definition (10, 11).

**World Health Organization definitions**

In the World Health Organization-Integrated Management of Childhood Illnesses, WHO-IMCI, sepsis is a diagnosis of exclusion, defined as presence of acute fever (>39°C) and severe illness when no other cause is found (12), while septic shock includes cold hands with poor peripheral perfusion; increased capillary refill time (>3 s); fast and weak pulse volume; hypotension; and decreased mental status (lethargy) (12).

**American College of Critical Care Medicine clinical practice parameters for hemodynamic support of pediatric and neonatal septic shock (2017 update)**

The American College of Critical Care Medicine defines sepsis as presence of hypothermia or hyperthermia plus clinical signs of inadequate tissue perfusion including any of the following: decreased or altered mental status; capillary refill time >2 s, diminished pulses, mottled cool extremities (cold shock); flash capillary refill, bounding peripheral pulses, wide pulse pressure (warm shock); urine output < 1 ml/kg/h. Hypotension is not necessary for clinical diagnosis of septic shock, but its presence in a child with clinical suspicion of infection is confirmatory (14).

**Fluid Expansion as Supportive Therapy definitions**

The Fluid Expansion As Supportive Therapy, FEAST, trial recruitment criteria required presence of the following: 1) fever (axillary body temperature >37.5 or < 36°C); 2) impaired consciousness (prostration or coma) and/or respiratory distress (increased work of breathing); 3) impaired perfusion (evidenced by one or more of the following criteria: capillary refill time of 3 or more seconds, lower limb temperature gradient, weak radial pulse volume, or severe tachycardia >180 beats per minute in children younger than 12 months of age, >160 beats per minute in children 1–5 years of age, or >140 beats per minute in children older than 5 years of age) (15).

**Table 1:** Selected age-specific variables are compared between different criteria to recognize sepsis and septic shock in children.

**Variable**	**Cut-off values (per age-group)**					
		**Heart rate (beats/min)**		**Respiratory rate (breaths/min)**	**Leucocyte count (WBC X 10^**9**^/L)**	**Systolic Blood Pressure (mm Hg)**
**Age-group classification**	**Age**	**Tachycardia**	**Bradycardia**	**Tachypnoea**	**Leucocytosis or leucopenia**	**Hypotension (11)**
**(A**) **Pediatric Sepsis Consensus Conference 2005 (PSCC) Criteria** (10).						
•Newborns	0 days−1 week	>180	< 100	>50	>34	<59
•Neonates	1 week−1 month	>180	<100	>40	>19.5 or < 5	<79
•Infant	1 month−1 year	>180	<90	>34	>17.5 or < 5	<75
•Toddler and pre-school	2–5 years	>140	N/A	>22	>15.5 or < 6	<74
•School age child	6–12 years	>130	N/A	>18	>13.5 or < 4.5	<83
•Adolescent and young adult	13 < 18 years	>110	N/A	>14	>11 or < 4.5	<90
Temperature** (hyper- or hypothermia)	> 38.5 or < 36.0 °C					
Prolonged capillary refill time	> 5 s					
**Variable**	**Cut-off values (per age-group)**					
**(B) American College of Critical Care Medicine (ACCM)** ^**##**^ (13, 14).						
Temperature (hyper- or hypothermia)	No cut-off values described					
Urine output	< 1 ml/kg/h					
Mental status	Decreased or altered mental status					
Capillary refill time	Prolonged >2 s (cold shock) or flash capillary refill (warm shock)					
Pulses	Diminished pulses and mottled cool extremities (cold shock) or bounding peripheral pulses with wide pulse pressure (warm shock)					
**Variable**	**Cut-off values (per age-group)**					
		**Heart rate (beats/min)**		**Systolic BP (mm Hg)**	**Respiratory rate (breaths/min)**^**$$**^	
**Age-group classification**	**Age**	**Tachycardia**	**Bradycardia**	**Hypotension**	**Tachypnoea**	**Bradypnoea**
**(C) World Health Organization-Integrated Management of Childhood Illnesses (WHO-IMCI) criteria** (12).						
	0 ≤ 1 year	>160	<100	<60	≥60	<20
	> 1 year ≤ 3 years	>150	<90	<70	≥50	<20
	> 3 years ≤ 6 years	>140	<80	<75	≥40	<20
Temperature (hyperthermia)	>39.0°C					
Prolonged capillary refill time	>3 s					
Hypoxia (SPO_2_)	< 90%					

** Temperature cut-off values apply for all ages and are based on core temperature measured by rectal, bladder, oral, or central catheter probe. ## Clinical diagnosis of shock based on ACCCM criteria requires suspected infection manifested by hypothermia or hyperthermia, and any of the above-listed clinical signs of inadequate tissue perfusion. $$ Respiratory rate criteria based on slightly different age-group classification cut-off values (i.e., < 2months, 2–11 months and 1–5 years).

## Changing Definitions, Unchanging Treatment

The Surviving Sepsis Campaign (SSC) guidelines focus on antibiotics, fluids, and inotropes as key elements of initial resuscitation (16). In the 2018 update of the adult Surviving Sepsis Campaign (SSC) bundle, a 1 h sepsis bundle for immediate management of sepsis is described, combining elements from previous 3 and 6 h bundles (17). The 1 h sepsis bundle makes a strong recommendation of administering 30 ml/kg bolus of crystalloid for resuscitation of adults with hypotension or lactate ≥4 mmol/L but further grades this recommendation as low quality given the available supporting evidence (17). In children, the recent ACCM recommendations advocate for administration of appropriate antibiotics, fluid boluses of up to 60 ml/kg, followed by initiation of inotropic support all within < 60 min, ideally within as little as 15 min, in children with septic shock (14). While there is supportive retrospective evidence for the recommendations of the 1 h sepsis bundle in children highlighting the need for early sepsis recognition, sampling for blood cultures, and administration of broad spectrum antibiotics (18), several components of the recognition and resuscitation bundles are based on expert opinion rather than evidence. Administration of rapid fluid boluses remains a cornerstone of treatment of shock, but the potential for harm related to large volume fluid administration is increasingly considered.

In 2011, the Fluid Expansion As Supportive Therapy (FEAST) multi-center randomized clinical trial (15) used pragmatic clinical and age-specific criteria for pediatric sepsis. These criteria in the FEAST trial were designed to generate practical, evidence-based data for management of children with severe febrile illness and impaired perfusion in resource-poor settings in sub-Saharan Africa and included over 3,000 patients (15). The landmark FEAST trial demonstrated that fluid boluses significantly increased 48 h mortality in acutely ill children with impaired perfusion in the resource-limited settings in South Saharan Africa (15, 19). A recent animal model of hyperdynamic endotoxaemic shock (20) reported paradoxical higher vasopressor requirement to maintain mean arterial blood pressure (MAP) following fluid bolus resuscitation which may account for some of the pathophysiology underlying findings in the FEAST study (21). More recently, the use of fluid boluses in septic shock in both pediatric (22) and adult populations (23, 24) is undergoing evaluation in several randomized controlled trials (Restrictive Intravenous Fluids Trial in Sepsis, RIFTS; Crystalloid Liberal or Vasopressors Early Resuscitation in Sepsis, CLOVERS).

In the 2016 update of the WHO pediatric emergency triage, assessment and treatment (ETAT) guideline, despite a search of 1,600 references, including 3 randomized controlled trials (RCTs), only the FEAST trial met the inclusion criteria for consideration regarding pediatric sepsis and septic shock (25).

*Post hoc* and pre-specified sub-group analyses of the FEAST trial suggested that the excess mortality observed with fluid bolus therapy was not attributable to factors such as under-recognition of fluid overload, high prevalence of malaria (57%) and severe anemia (hemoglobin levels below 5 g/dL in 32%) in the study population; indeed fluid boluses were associated with adverse outcomes in all sub-groups analyzed (19, 26).

In contrast to adults, where large trials were performed on key interventions such as the use of hydrocortisone in septic shock (27), or on the use of norepinephrine and dopamine in septic shock (28), there are no comparably powered pediatric trials published or ongoing. Currently, to the best of our knowledge there are no large randomized controlled trials ongoing which compare fluid bolus therapy with alternative interventions such as vasopressors, or steroids in pediatric patients with septic shock (29), resulting in ongoing controversy around best practice.

In the past decade, outside the FEAST trial, only a relatively small number of interventional trials in pediatric sepsis were conducted, the majority of those with < 100 included patients (30–33). In 2008, Santhanam et al. found no differences in mortality or resolution of shock when comparing resuscitation of 147 children with septic shock using 40 mls/kg fluid over 15 min followed by dopamine vs. 20 mls/kg fluid over 20 min up to a maximum of 60 mls/kg/h followed by dopamine (34). Oliveira et al. observed reduced mortality when using superior vena cava oxygenation as an end-point in goal-directed therapy in children with septic shock (35). A more recent trial from the United Kingdom highlighted challenges pertinent to feasibility of trials investigating the volume of fluid resuscitation in sepsis (22).

Future research on optimal hemodynamic support in sepsis and septic shock should consider assessing the role of volume, type (balanced crystalloids vs. normal saline) (36, 37), rate, and temperature of fluids and evaluate fluid-sparing strategies such as early vasoactive and inotrope support. Studies should include sites in resource-limited settings *inter alia* (26), as well as addressing and adjusting for variations attributable to different settings pertinent to host and pathogen characteristics all of which are likely to affect susceptibility, response, and outcomes (38).

In conclusion, it is urgent that the pediatric community collaborates across the globe to address the need for meaningful, pertinent and harmonized sepsis criteria that can be applied to children in different settings, including in- and outside intensive care, both in high-income countries and LMIC. This will allow the rigorous evaluation of the impact of sepsis bundles. Robust criteria will facilitate design and recruitment into novel trials to improve the evidence for currently recommended treatments to result in improved outcomes for children with sepsis.

## Author Contributions

All authors listed have made a substantial, direct and intellectual contribution to the work, and approved it for publication.

### Conflict of Interest Statement

The authors declare that the research was conducted in the absence of any commercial or financial relationships that could be construed as a potential conflict of interest.
