# Corrigendum: Sepsis: Changing Definitions, Unchanging Treatment

**DOI:** 10.3389/fped.2019.00538

**Published:** 2020-01-23

**Authors:** Nchafatso Gikenyi Obonyo, Luregn Jan Schlapbach, John Francis Fraser

**Affiliations:** ^1^Initiative to Develop African Research Leaders (IDeAL-DELTAS), Kilifi, Kenya; ^2^KEMRI-Wellcome Trust Research Programme, CGMRC, Kilifi, Kenya; ^3^Critical Care Research Group, The Prince Charles Hospital, Chermside, QLD, Australia; ^4^Wellcome Trust Centre for Global Health Research, Imperial College London, London, United Kingdom; ^5^Paediatric Critical Care Research Group, Child Health Research Centre, The University of Queensland, South Brisbane, QLD, Australia; ^6^Paediatric Intensive Care Unit, Queensland Children's Hospital, South Brisbane, QLD, Australia; ^7^Department of Pediatrics, Inselspital Universitätsspital Bern, Bern, Switzerland; ^8^Faculty of Medicine, The University of Queensland, Herston, QLD, Australia; ^9^School of Medicine, Griffith University, Gold Coast, QLD, Australia

**Keywords:** sepsis, septic shock, definitions, pediatric populations, treatment bundles

In the original article, we neglected to include the funders “DELTAS Africa Initiative,” “DEL-15-003” to “Nchafatso Gikenyi Obonyo” and the “AAS, NEPAD Agency, the Wellcome Trust and the UK government.”

A correction has been made to the **Funding** statement:

“NO was supported and funded through the DELTAS Africa Initiative [DEL-15-003]. The DELTAS Africa Initiative is an independent funding scheme of the African Academy of Sciences (AAS)'s Alliance for Accelerating Excellence in Science in Africa (AESA) and supported by the New Partnership for Africa's Development Planning and Coordinating Agency (NEPAD Agency) with funding from the Wellcome Trust [107769/Z/10/Z] and the UK government. The views expressed in this publication are those of the author(s) and not necessarily those of AAS, NEPAD Agency, Wellcome Trust or the UK government.

This paper was supported through the Critical Care Research Group (CCRG) and the Paediatrics Critical Care Research Group (PCCRG), Brisbane, Australia.”

Furthermore, in the published article, there was an error in affiliation 1. Instead of “IDeAL/KEMRI-Wellcome Trust Research Programme, CGMRC, Kilifi, Kenya,” it should be “Initiative to Develop African Research Leaders (IDeAL-DELTAS), Kilifi, Kenya” and “KEMRI-Wellcome Trust Research Programme, CGMRC, Kilifi, Kenya.”

The affiliation list has been updated to reflect the additional affiliation.

The authors apologize for these errors and state that this does not change the scientific conclusions of the article in any way. The original article has been updated.

